# Impact of Delayed Pain to Needle and Variable Door to Needle Time On In-Hospital Complications in Patients With ST-Elevation Myocardial Infarction Who Underwent Thrombolysis: A Single-Center Experience

**DOI:** 10.7759/cureus.21205

**Published:** 2022-01-13

**Authors:** Arshad Muhammad Iqbal, Syed Farrukh Jamal, Adnan Ahmed, Hassan Khan, Waqar Khan, Faisal Ahmed, Ramchandani Santosh, Muhammad Salman Ghazni, Ateeq Mubarik, Bashir Hanif

**Affiliations:** 1 Department of Cardiovascular Medicine, University of Missouri School of Medicine, Columbia, USA; 2 Internal Medicine, Oak Hill Hospital, Brooksville, USA; 3 Cardiology, Tabba Heart Institute, Karachi, PAK; 4 Cardiology, Cleveland Clinic Abu Dhabi, Abu Dhabi, ARE; 5 Internal Medicine, Saint Joseph Hospital, Chicago, USA; 6 Cardiology, National Institute of Cardiovascular Diseases, Karachi, PAK; 7 Cardiology, Dow University of Health Sciences, Civil Hospital Karachi, Karachi, PAK; 8 Internal Medicine, Nephrology, Lehigh Valley Hospital, Allentown, USA; 9 Sleep Medicine, New York Sleep Disorder Center, Brooksville, USA; 10 Internal Medicine, Ascension St. Michael's Hospital, Stevens Point, USA

**Keywords:** thrombolysis, stemi, in-hospital complications, delayed door to needle time, delayed pain to needle time

## Abstract

Background

Myocardial infarction is a life-threatening event, and timely intervention is essential to improve patient outcomes and mortality. Previous studies have shown that the time to thrombolysis should be less than 30 minutes of the patient’s arrival at the emergency room. Pain-to-needle time is a time from onset of chest pain to the initiation of thrombolysis, and door-to-needle time is a time between arrival to the emergency room to initiation of thrombolytic treatment. Ideally, the target for door-to-needle time should be less than 30 minutes; however, it is unclear if the door-to-needle time has a significant impact on patients presenting later than three hours from the onset of pain. As many of the previous studies were conducted in first-world countries, with established emergency medical services (EMS) systems and pre-hospital ST-elevation myocardial infarction (STEMI) triages and protocols, the data is not completely generalizable to developing countries. We, therefore, looked for the impact of the shorter and longer door-to-needle times on patient outcomes who presented to the emergency room (ER) with delayed pain-to-needle times (more than three hours of pain onset).

Objective

To determine the impact of delayed pain-to-needle time (PNT) with variable door-to-needle time (DNT) on in-hospital complications (post-infarct angina, heart failure, left ventricular dysfunction, and death) in patients with ST-elevation myocardial infarction (STEMI) who underwent thrombolysis.

Methods and results

A total of 300 STEMI patients who underwent thrombolysis within 12 hours of symptoms onset were included, which were divided into two groups based on PNT. These groups were further divided into subgroups based on DNT. The primary outcome was in-hospital complications between the two groups and between subgroups within each group. The pain-to-needle time was ≤3 hours in 73 (24.3%) patients and >3 hours in 227 (75.7%) patients. In-hospital complications were higher in group II with PNT >3 hours (p <0.05). On subgroup analysis, in-hospital complications were higher with longer door-to-needle time in group II (p<0.05); however, there was no difference in complications among group I.

Conclusion

Our study is consistent with the fact that shorter door-to-needle time, even in patients with delayed PNT (>3 hours), has a significant impact on in-hospital complications with no difference in mortality.

## Introduction

Restoration of blood flow to the culprit artery is the main principle for the treatment of acute ST-segment elevation myocardial infarction (STEMI). This has been proven to decrease the size of infarction and improve overall survival; however, according to Global Registry of Acute Coronary Events (GRACE) data, up to 40% of patients presenting acutely with STEMI fail to receive appropriate reperfusion therapy [1].

Timely reperfusion of ischemic myocardium is the cornerstone in the management of STEMI. The latest American & European guidelines suggested a “door-to-needle time” (DNT), which is from arrival to the hospital till initiation of thrombolytic injection should be less than 30 minutes, is taken as the current gold standard and quality metric for timely reperfusion via pharmacological thrombolysis [2].

The “pain-to-needle time’’ (PNT), which is the time taken from the onset of chest pain till the administration of the thrombolytic injection, represents the total time the artery was occluded, and the myocardium was deprived of blood flow. Mortality reduction can be up to 50% if thrombolysis is started within 180 minutes, i.e., three hours of symptom onset [3]. This thrombolytic benefit can be retained for up to 12 hours in certain cases where there is evidence of ongoing active ischemia [4].

As per GRACE data, almost up to 40% of patients present late and end up missing this time window for thrombolytic therapy [5]. All the available data and the current guidelines emphasize DNT regarding reperfusion strategies with most of the studies done in the western population. This delay in reperfusion therapy leads to an increased risk of associated complications of acute myocardial infarction such as post-infarct angina, heart failure, left ventricular dysfunction, and death [6]; however, real-world data for developing countries is extremely limited. Due to lack of resources (such as unavailability of an air ambulance to commute, pre-hospital thrombolysis) and delay in seeking help, as these factors may have a significant role in putting at risk for significant complications and mortality, it is important to determine if the door-to-needle time still has a significant role and if this would impact in-hospital outcomes in patients who present more than three hours of STEMI onset.

## Materials and methods

We performed a single-center retrospective cohort study by extracting information from institutional data of patients hospitalised for STEMI between July 2015 and December 2015. A total of 300 adult patients who underwent thrombolysis for the first presentation of ST-elevation myocardial infarction within 12 hours of symptoms onset were included. The patients with a previous history of thrombolysis, prior myocardial infarction (MI), heart failure with Killip class IV, and prior LV dysfunction documented on the previous echocardiogram were excluded.

The data recorded comprised of patient's demographics, Electrocardiogram (ECG) changes, pain-to-needle time (PNT), door-to-needle time (DNT), and in-hospital complications (post-MI angina, heart failure, LV dysfunction, and death) and echocardiographic parameters. Outcomes were assessed based on chart review and documentation on hospital discharge. Permission from the institutional review board (IRB) was obtained before the commencement of the study. 

**Figure 1:**
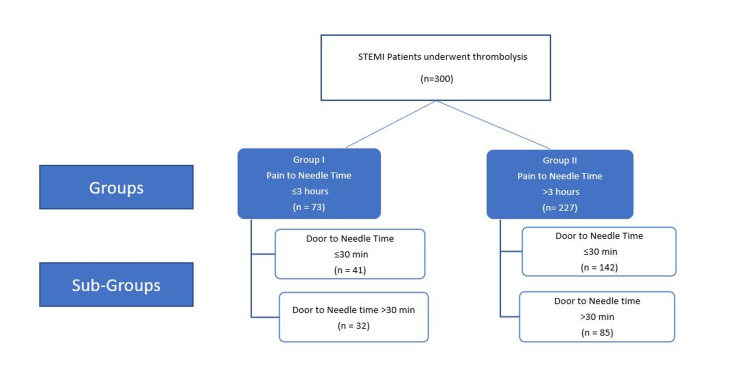
Classification of Groups and Sub-groups

The patients were divided into two groups based on early and delayed PNT (Group I: early PNT with PNT ≤3 hours; Group II: delayed PNT with PNT 3-12 hours). Each group was further sub-divided based on shorter (≤30 min) vs. longer (>30min) DNT (Figure 1). In-hospital complications (post-infarct angina, heart failure, left ventricular dysfunction, and death) were compared between the two groups as well as between subgroups within each group.

The primary outcomes of the study were to compare the in-hospital complications between groups I and II and within each subgroup. Complication rates were calculated as frequencies and percentages. Post-MI angina was defined as new chest pain after the resolution of the initial chest pain episode. Heart failure was defined as the development of new shortness of breath with New York Heart Association functional classification I-III. Left ventricular dysfunction was defined as an ejection fraction of less than 50% on echocardiographic documentation. Left ventricular dysfunction was considered mild if ejection fraction (EF) between 35%-50%, moderate if EF between 25%-35%, and severe if EF was less than 25%. 

IBM Corp. Released 2015. IBM SPSS Statistics for Windows, Version 23.0. Armonk, NY: IBM Corp. was used for data entry and analysis. Mean and standard deviation were calculated for age, pain-to-needle, and door-to-needle times. Frequency and percentages were calculated for gender, diabetes, hypertension, smoker, delayed DNT, delayed PNT, and in-hospital complications. The Chi-square test was used for qualitative variables. P-value ≤ 0.05 was taken as significant.

## Results

Table 1 shows the baseline characteristics of the patients with early and delayed PNT groups.

**Table 1:** Baseline characteristics

Characteristics (n=300)	Pain-to-needle time ≤ 3 hours (n =73) (Group I)	Pain-to-needle time 3-12 hours (n =227) (Group II)	P-Value
Age			0.015
≤ 55 years (154)	53	101	
>55 years (146)	20	126	
Gender			0.464
Male (266)	63	203	
Female (34)	10	24	
Diabetes			0.001
Yes (126)	9	117	
No (174)	64	110	
Hypertension			0.001
Yes (144)	22	122	
No (156)	51	105	
Smoking			0.001
Yes (178)	60	118	
No (122)	13	109	
Obesity			0.012
Yes (53)	20	33	
No (247)	53	194	

There were 266 male and 34 female patients included in the study. The mean age of study subjects was 54.99±7.74 years, with the mean duration of chest pain being 196.15±87.85 minutes. Out of all patients, 42.0% of the patients were diabetic, 48.0% were hypertensive, 59.3% were smokers, and 17.7% were obese.

The pain-to-needle time of 73 patients was ≤3 hours (group I; early PNT group), while it was 3-12 hours in 227 patients (group II; delayed PNT group). Group I and II were further divided into shorter (≤30 minutes) and longer DNT (>30 minutes) times. In group I, shorter DNT was observed in 41 patients, while longer DNT was observed in 32 patients. In group II, shorter DNT was observed in 142 patients, while longer DNT was observed in 85 patients. See Figure 1.

There was a significant association of pain-to-needle time observed with age (p<0.0001), diabetes mellitus (p<0.0001), hypertension (p<0.0001), smoking, obesity (p=0.012) (Table 1).

In-hospital complications were compared based on early and delayed PNT groups. When comparing groups I and II (PNT ≤3 vs. 3-12 hours), post-infarct angina was observed in 2.7% in group I vs. 22.9% in group II with a P-value of 0.001. Heart failure with Killip II was observed in 8.2% vs. 11.45%, while heart failure with Killip III was observed in 0% vs. 7.9% (P=0.001). Mild LV dysfunction was observed in 12.3% in group I vs. 9.6% in group II, moderate LV dysfunction in 5.4% vs. 57%, severe LV dysfunction in 2.7% vs. 5.7% (P value 0.001). Death was observed in 0% in group I while 2.2% in group II with a P-value of 0.340. Therefore, there was a significant difference in post-infarct angina, heart failure, and LV dysfunction when thrombolytics were given within three hours of pain, with no difference in death between these groups. (Table 2).

**Table 2:** Comparison of outcomes between groups I and II.

Outcomes (n=300)	Pain-to-Needle Time ≤ 3 hours (n =73) (Group I)	Pain-to-Needle Time 3-12 hours (n =227) (Group II)	P-Value
Post-infarct Angina			<0.0001
Yes (54)	2	52	
No (246)	71	175	
Heart Failure			<0.0001
Killip I (250)	67	183	
Killip II (32)	6	26	
Killip III (18)	0	18	
Left Ventricular Dysfunction			<0.0001
No (119)	58	61	
Mild (31)	9	22	
Moderate (135)	4	131	
Severe (15)	2	13	
Death			0.340
Yes (5)	0	5	
No (295)	73	222	

In-hospital complications were further compared within each PNT group based on DNT as mentioned above (Figure 1). In group I, there was no significant difference in post-MI angina, heart failure, LV dysfunction, and death with shorter vs. longer door to needle time (Table 3).

**Table 3:** Comparison of outcomes based on shorter and longer door-to-needle time in group I NS: non-significant (p > 0.05)

Outcomes (n=73)	Pain-to-Needle Time ≤ 3 Hours (n =73)		P-Value
	Door-to-Needle Time ≤ 30 min (n=41)	Door to Needle Time > 30 min (n=32)	
Post-infarct Angina			NS
Yes (2)	0	2	
No (71)	41	30	
Heart Failure			NS
Killip I (67)	39	28	
Killip II (6)	2	4	
Killip III (0)	0	0	
Left Ventricular Dysfunction			NS
No (58)	37	21	
Mild (9)	3	6	
Moderate (4)	1	3	
Severe (2)	0	2	
Death			NS
Yes (0)	0	0	
No (73)	41	32	

In group II, when outcomes were compared based on shorter vs. longer door-to-needle time; post-infarct angina, heart failure, and LV dysfunction were observed more in the longer door-to-needle time, as compared to shorter door-to-needle time with a significant P value of less than 0.05. However, there was no significant difference in death among group II. (Table 4).

**Table 4:** Comparison of outcomes based on shorter and longer door to needle time in Group II

Outcomes (n=227)	Pain-to-Needle Time 3-12 Hours (n =227)		P-Value
	Door to needle time ≤30 min (n=142)	Door to needle time > 30 min (n=85)	
Post-infarct angina			<0.0001
Yes (52)	4	48	
No (175)	138	37	
Heart Failure			0.001
Killip I (183)	136	47	
Killip II (26)	4	22	
Killip III (18)	2	16	
Left Ventricular Dysfunction			<0.0001
No (61)	59	2	
Mild (22)	15	7	
Moderate (131)	66	65	
Severe (13)	2	11	
Death			0.340
Yes (5)	2	3	
No (222)	140	82	

## Discussion

Acute myocardial infarction (AMI) is managed on the guiding principle of restoring blood supply to the ischemic myocardium, which decreases morbidity and mortality, and primary percutaneous coronary intervention (PPCI) is the treatment of choice in this setting. As evidenced by multiple large trials, up to a third of these patients with AMI don’t get these timely lifesaving interventions, and up to two-thirds of these patients are those that seek help but initially present to setups that don’t have the facility of performing PPCI [7,8]. In the western world, established point of care systems and emergency services help in providing access to PPCI for almost 80% of such patients [9]. However, in the developing world, with limited emergency services and resources and a limited number of PPCI capable facilities, this timely access, and urgent management is even a bigger problem. Even in PPCI capable facilities, the cost burdens are also significant and create hurdles in the delivery of care, and this has made pharmacologic thrombolysis a crucial step in acute management.

The European Society of Cardiology emphasizes timely reperfusion such that they came up with the famous quote “time is muscle”. Therefore, the survival benefit is governed by an entire chain of events starting from early recognition of symptoms to the prompt delivery of emergency services (EMS) and prompt transfer to a PPCI capable hospital [10,11]. Multiple studies, including the Zagreb study and Castiella et al., concluded that the biggest factor in pre-hospital delay was late recognition of symptoms and hence delay in seeking care, and this was independent of the effectiveness of the team or availability of the emergency services [12,13]. This observation was also shown in our study by the mean duration of chest pain being well over three hours. The missed diagnosis of the pain as GERD symptoms, ignorance, denial, and lack of enough facilities could be the reason for the late arrival time in our study. Studies have shown that pain-to-call time, also known as “decision time”, and its prolongation correlates well with patient factors which include the nocturnal onset of symptoms, higher pain tolerance, rural origin, and history of diabetes [14,15]. Women and the elderly have also seen delays in seeking help [16]. Studies have also demonstrated that reaching out to primary care physicians in such cases also adds to pre-hospital delay [17].

Pre-hospital thrombolysis can serve as a viable alternative when PPCI isn’t possible within 90 minutes. A caveat here is the availability of advanced cardiac life support (ACLS)-equipped ambulances and trained staff for administering these therapies and monitoring their effects. Abba et al. showed that thrombolytic therapy was administered to the majority of patients presenting with acute MI within two hours of their hospital arrival [3]. A study from Finland demonstrated that only 38% of patients received thrombolysis within two hours of symptom onset [18]. Delay in thrombolysis may be due to time elapsed for evaluation of patients in the emergency department (ED) or until referral to coronary care unit (CCU) where thrombolysis can be administered [19]. In-hospital factors accounted for up to 59% of the delays from symptom onset to thrombolytic administration. After arrival in the emergency room, an average of 20 minutes were required to obtain an EKG and further 70 minutes before the administration of thrombolysis [20]. Initiating thrombolysis in the ED rather than CCU also reduced time delays by as much as 60 minutes [21].

Prompt thrombolysis and CCU care within one-and-a-half hours of symptom onset translate into smaller infarcts sizes, more preservation of left ventricular function, and lower 21-day mortality [22]. The results of the Grampian Region Early Anistreplase Trial (GREAT trial) comparing pre-hospital with in-hospital thrombolysis and favored the prehospital group where patients received thrombolytic treatment more than two hours earlier (101 versus 240 minutes after the onset of symptoms) and had up to a 50% risk reduction in annual mortality [23]. This benefit was consistent even at a five-year follow-up, and the mortality in the prehospital treated group was 25% compared to 36% in the hospital treated patients. Another meta-analysis comprising of six randomized trials involving 6,434 patients showed a significant reduction in all-cause mortality in prehospital thrombolysis compared to in-hospital thrombolysis [24].

Our observations are also in line with older studies mentioned above that there is no difference in mortality outcomes in early arrivals, i.e., within three hours. However, prompt administration of thrombolysis within 30 minutes of arrival in late arrivals, i.e., more than three hours, did show a significant trend towards less post-infarct angina, less LV dysfunction, and less heart failure. We, therefore, conclude that DNT should take precedence in improving outcomes on in-hospital complications in late arrivals. However, we did not find any significant difference in mortality between either of the groups.

Study limitations

This is a retrospective study and information based solely on chart documentation. Our study is limited by its single location in an urban environment. We only included those patients who mentioned chest pain with ST-elevation MI, and possibly we could have missed patients with acute coronary syndrome (ACS) equivalent symptoms such as shortness of breath, weakness, and neck/shoulder/back pain. Enzymatic infarct size was not available in all patients, with a potential underestimation of the impact of early treatment on myocardial salvage.

## Conclusions

The results indicate that even with the delay in presentation, rapid administration of thrombolytic therapy improves in-hospital outcomes. Any delay in door-to-needle or pain-to-needle time in STEMI patients was associated with increased in-hospital complications. Thus, reducing this time delay to the greatest extent should be made possible for such patients. A collaborative approach between the Emergency Department and the cardiology team and better prehospital triage and transfer services can improve delays and potentially improve outcomes. In a resource-limited country, all efforts should be made to decrease these delays to provide better patient care and increase chances of survival.

## References

[1] Thygesen K, Alpert JS, Jaffe AS, Chaitman BR, Bax JJ, Morrow DA, White HD (2018). Fourth universal definition of myocardial infarction (2018). Circulation.

[2] Welsh RC, Ornato J, Armstrong PW (2003). Prehospital management of acute ST-elevation myocardial infarction: a time for reappraisal in North America. Am Heart J.

[3] Abba AA, Wani BA, Rahmatullah RA, Khalil MZ, Kumo AM, Ghonaim MA (2003). Door to needle time in administering thrombolytic therapy for acute myocardial infarction. Saudi Med J.

[4] White HD, Van de Werf FJ (1998). Thrombolysis for acute myocardial infarction. Circulation.

[5] Chotechuang Y, Phrommintikul A, Muenpa R (2016). The prognostic utility of GRACE risk score in predictive cardiovascular event rate in STEMI patients with successful fibrinolysis and delay intervention in non PCI-capable hospital: a retrospective cohort study. BMC Cardiovasc Disord.

[6] Lambert L, Brown K, Segal E, Brophy J, Rodes-Cabau J, Bogaty P (2010). Association between timeliness of reperfusion therapy and clinical outcomes in ST-elevation myocardial infarction. JAMA.

[7] Reddy K, Khaliq A, Henning RJ (2015). Recent advances in the diagnosis and treatment of acute myocardial infarction. World J Cardiol.

[8] Frans Van de Werf, Diego Ardissino, Amadeo Betriu (2003). Management of acute myocardial infarction in patients presenting with ST-segment elevation. Eur Her J.

[9] Daniels MJ, Cohen MG, Bavry AA, Kumbhani DJ (2020). Reperfusion of ST-segment-elevation myocardial infarction in the COVID-19 era: business as usual?. Circulation.

[10] (2018). Corrigendum. Eur Heart J.

[11] Ornato JP (2007). The ST-segment-elevation myocardial infarction chain of survival. Circulation.

[12] Ivanuša M, Verica K, Olivari M (2019). Mortality from ischemic heart disease and acute myocardial infarction in the city of Zagreb and Republic of Croatia 2001-2016. Cardiol Croat.

[13] Castiella J, Valdearcos S, Alquezar ML (1997). Analysis of causes for an excessive prehospitalization delay in patients with acute myocardial infarction in teruel (SPAIN). Rev Esp Cardiol.

[14] Nielsen CG, Laut KG, Jensen LO, Ravkilde J, Terkelsen CJ, Kristensen SD (2017). Patient delay in patients with ST-elevation myocardial infarction: time patterns and predictors for a prolonged delay. Eur Hr J Acu Cardiov Care.

[15] Johansson I, Strömberg A, Swahn E (2004). Factors related to delay times in patients with suspected acute myocardial infarction. Hea Lu.

[16] McSweeney JC, Rosenfeld AG, Abel WM (2016). Preventing and experiencing ischemic heart disease as a woman: state of the science: a scientific statement from the American Heart Association. Circulation.

[17] Hitchcock T, Rossouw F, McCoubrie D, Meek S (2003). Observational study of prehospital delays in patients with chest pain. Emerg Med J.

[18] Hirvonen TP, Halinen MO, Kala RA, Olkinuora JT (1998). Delays in thrombolytic therapy for acute myocardial infarction in Finland: Results of a national thrombolytic therapy delay study. Eur Heart J.

[19] Alishahi Tabriz A, Sohrabi MR, Kiapour N, Yazdani S (2012). Factors associated with delay in thrombolytic therapy in patients with ST-elevation myocardial infarction. J Tehran Hea Cent.

[20] Bassand JP, Danchin N, Filippatos G (2005). Implementation of reperfusion therapy in acute myocardial infarction. A policy statement from the European Society of Cardiology. Eur Heart J.

[21] Beig JR, Tramboo NA, Kumar K (2017). Components and determinants of therapeutic delay in patients with acute ST-elevation myocardial infarction: a tertiary care hospital-based study. J Saudi Heart Assoc.

[22] Bagai A, Dangas GD, Stone GW, Granger CB (2014). Reperfusion strategies in acute coronary syndromes. Circ Res.

[23] Rawles JM (1997). Quantification of the benefit of earlier thrombolytic therapy: five-year results of the Grampian Region Early Anistreplase Trial (GREAT). J Am Coll Cardiol.

[24] Morrison LJ, Verbeek PR, McDonald AC, Sawadsky BV, Cook DJ (2000). Mortality and prehospital thrombolysis for acute myocardial infarction: a meta-analysis. JAMA.

